# Quantifying Tubulin Concentration and Microtubule Number Throughout the Fission Yeast Cell Cycle

**DOI:** 10.3390/biom9030086

**Published:** 2019-03-04

**Authors:** Isabelle Loiodice, Marcel E. Janson, Penny Tavormina, Sebastien Schaub, Divya Bhatt, Ryan Cochran, Julie Czupryna, Chuanhai Fu, Phong T. Tran

**Affiliations:** 1 Cell & Developmental Biology, University of Pennsylvania, Philadelphia, PA 19104, USA; 2 Molecular Devices Corporation, Downingtown, PA 19335, USA; 3 Institut Curie, PSL Research University, CNRS, UMR 144, F-75005 Paris, France

**Keywords:** microtubule dynamics, spindle morphogenesis

## Abstract

The fission yeast *Schizosaccharomyces pombe* serves as a good genetic model organism for the molecular dissection of the microtubule (MT) cytoskeleton. However, analysis of the number and distribution of individual MTs throughout the cell cycle, particularly during mitosis, in living cells is still lacking, making quantitative modelling imprecise. We use quantitative fluorescent imaging and analysis to measure the changes in tubulin concentration and MT number and distribution throughout the cell cycle at a single MT resolution in living cells. In the wild-type cell, both mother and daughter spindle pole body (SPB) nucleate a maximum of 23 ± 6 MTs at the onset of mitosis, which decreases to a minimum of 4 ± 1 MTs at spindle break down. Interphase MT bundles, astral MT bundles, and the post anaphase array (PAA) microtubules are composed primarily of 1 ± 1 individual MT along their lengths. We measure the cellular concentration of αβ-tubulin subunits to be ~5 µM throughout the cell cycle, of which one-third is in polymer form during interphase and one-quarter is in polymer form during mitosis. This analysis provides a definitive characterization of αβ-tubulin concentration and MT number and distribution in fission yeast and establishes a foundation for future quantitative comparison of mutants defective in MTs.

## 1. Introduction

The fission yeast *Schizosaccharomyces pombe* serves as a good genetic model organism for investigating diverse cellular processes such as cell cycle and cell morphogenesis [1,2]. Fission yeast is also a good organism for quantitative dynamic imaging studies of fluorescently tagged proteins [3,4]. Fluorescence imaging has revealed the cellular concentration of actin and actin-associated proteins in fission yeast [3,4]. Similar quantifications for microtubules (MTs) and associated-proteins are lacking. Processes such as MT dynamics and organization during interphase and mitosis have been dissected using fluorescent live cell imaging [5,6,7,8]. These studies described qualitatively the general organization and function of the MT cytoskeleton throughout the cell cycle. For example, imaging revealed that fission yeast has several different MT organizing centers (MTOCs). During interphase, the spindle pole body (SPB) and the multiple interphase MTOCs (iMTOCs) organize 3–5 antiparallel linear bundles of MTs [6,8]. Interphase MTs function in nuclear positioning by producing polymerization-dependent pushing forces to dynamically center the nucleus at the cell middle [8,9,10]. Interphase MTs also function to recruit polarity factors to the cell tips and, therefore, control the direction of cell growth and cell shape [11,12,13]. During mitosis, the SPBs organize the mitotic spindle for chromosomal segregation. The mitotic spindle has three distinct phases of elongation, corresponding to distinct stages of mitosis [14]. The SPBs also organize the astral MTs, which function similarly to interphase MTs in nuclear and spindle positioning [15]. At late mitosis, the equatorial MTOC (eMTOC) organizes the post-anaphase array (PAA) of MTs, which are responsible for maintaining the position of the acto-myosin contractile ring at the cell middle [15]. Mechanisms of assembly of these diverse MTOCs and MT arrays appear to involve the Mto1–Mto2 protein complex which activates MT nucleation [16,17,18]. Given its genetic tractability, relatively simple MT cytoskeleton and ease-of-use in imaging studies, we anticipate that a quantitative method which measures exact values of cellular tubulin concentration and/or MT number would greatly advance our understanding of mechanisms regulating MT nucleation, organization, and function. In particular, precise values of tubulin concentration and MT number would aid predictive modeling of MT-dependent processes.

Quantitative methods such as mass spectrometry and electron microscopy have been used to measure tubulin concentration and MT number and organization in fission yeast [19,20,21,22,23]. These methods lack time resolution representing dynamic changes. Nevertheless, they serve as important foundational work for comparison and interpretation of live-cell fluorescent imaging data. We describe here a simple quantitative fluorescent imaging and analysis method that has the resolution to count individual MTs in living fission yeast cells. We applied this method to measure MT number and distribution in wild-type cells throughout the cell cycle. We also present an in vivo measurement of the cellular αβ-tubulin concentration and define how tubulin is partitioned between soluble tubulin and MT polymer in the cell throughout the cell cycle.

## 2. Methods

### 2.1. Cell Strain and Preparation

Standard *S. pombe* techniques and media were used as previously described [24]. One fission yeast strain expressing GFP-Atb2 was used in this study (PT.47 h-leu1-32 + nmt1-GFP-Atb2). In preparation for live-cell imaging, cells were grown in 3 mL shaking cultures at 25 °C to optical density OD_600nm_ ~0.5. One milliliter of cells was then pelleted in a microfuge at 10,000 g for 15 s and then re-suspended in 100 µL of medium. One microliter volume of the cells was then placed in a sealed 2% agarose chamber as previously described [25]. Chambers were made fresh for each experiment. Cells were viable in the sealed chambers for several hours. Cells were imaged at room temperature, typically at 21–23 °C.

We made one important assumption in the current study: The use of the tubulin nmt1-GFP-Atb2 does not significantly alter MT numbers and dynamics in cells [5]. Previous studies using nmt1-GFP-Atb2 support this assumption. (1) The *nmt1* promotor is strongly repressed in the presence of 2μM thiamine [26]. The PT.47 strain was grown and imaged under 2 μM thiamine repression. Thus, the GFP-Atb2 expression level is anticipated to be low. (2) Cells expressing nmt1-GFP-Atb2 have no detectable phenotype in terms of the cell cycle, cell shape, mitosis, and MT organization compared to untagged wild-type cells [5,7,8]. (3) Our spindle MT measurement using nmt1-GFP-Atb2 matched very well with the electron microscopy (EM) measurement of spindle MTs in cells with native Atb2 expression [20].

### 2.2. Microscope Set-Up

Live cell imaging was performed with a spinning disk confocal microscope, which has been shown to create very low photobleaching [27]. We used the Yokogawa CSU-10 confocal scan head (www.yokogawa.com), attached to a Nikon E600FN upright microscope equipped with a PlanApo 100×/1.45NA DIC objective lens (www.nikonusa.com). An Argon-Krypton ion laser with ~10 mW power at 488 nm excitation illuminated the scanning unit through an optical fiber (www.mellesgriot.com). The effective laser power which reached the cells was ~0.2 mW. Images were captured with a cooled back-thinned ORCA-II-ER CCD (charge coupled device) camera (www.hamamatsu.com) controlled by MetaMorph 6.0 (www.moleculardevices.com) running on Windows XP.

### 2.3. Quantitative Imaging

**Figure 1:**
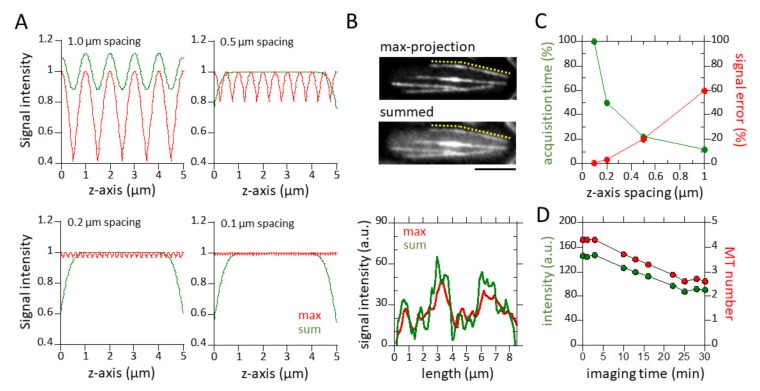
Practical parameters for quantitative fluorescence measurements. (**A**) Shown are normalized intensity plots at four different z-spacings (0.1, 0.2, 0.5 and 1 µm) of a 5-µm-thick section required to cover the complete thickness of a typical fission yeast cell (4 µm diameter). Intensity measurements at each z-spacing can yield an error if the object measured is out of focus. Error is highest when an object is half-way between two in-focus z-planes (red lines). The spacing between two z-planes determines the magnitude of measurement error, i.e., 0.1 µm z-spacing gives a 1% error, whereas 1 µm z-spacing gives a 40% error. Error approaches 0% when the intensities of all z-planes are summed (green lines). Note that at 1 µm z-spacing, the summed intensity began to show significant error. (See Methods). (**B**) Maximum-projection and summed images of a fission yeast cell expressing GFT-Atb2 (tubulin). Interphase MT bundles are shown. Yellow dotted lines approximate the MT bundle scanned. The accompanying bottom plot shows intensity line scans of the max-projected MT and the summed MT. (**C**) Shown is an optimization plot of z-spacing error versus total z-planes. The required total z-planes depends on the z-spacing and sample thickness, i.e., 51 planes are required at 0.1 µm spacing to cover a 5-µm-thick sample, whereas only six planes are required at 1 µm spacing to cover the same thickness. Correspondingly, the time to capture the required total planes also depends on the z-spacing, i.e., the time required for six planes is 12% of the time required for 51 planes. The intersection of the z-spacing error and total z-planes (time) yields the optimized z-spacing of 0.5 µm for a 5-µm-thick sample. (**D**) Plot showing photobleaching versus time. Under our imaging condition (see Methods), the cytoplasmic signal of GFP-Atb2 decreases ~40% after 30 min imaging time. This represents a counting error of ~2 MTs.

A typical fission yeast cell is rod-shaped, with a 4 μm diameter and 7–14 μm length depending on the cell cycle stage. To capture the complete MT cytoskeleton within the cell, we need to scan a 5-μm-thick section through the cell. In theory, if the MT is located exactly on one of the focused z-planes, then the intensity in that focused z-plane can be used as a measure of MT number. However, if the MT is located between two z-planes, then the intensity will appear to be lower, leading to measurement errors. To estimate the intensity error due to being outside the focused plane, we measured the point spread function (PSF) attributed to our microscope set-up. The PSF was measured by scanning a 0.02 μm diameter green fluorescence bead (probes.invitrogen.com) with a z-spacing or step size of 0.05 μm for a 5-μm-thick section. The shape of the PSF in the *z*-axis could be best fitted to a Gaussian profile with a full-width-half-maximum (FWHM) of 0.89 μm. The normalized intensity in a focused plane at a distance z away from the MT equals:

(1) $$I\left(z\right)=exp\left(-\frac{1}{2}{\left(z/\sigma \right)}^{2}\right)$$

where *σ* = 0.425 × FWHM = 0.378. Based on this equation, Figure 1A shows how much the measured intensity at various positions between the two focused planes differs from the normalized maximum intensity of 1 at the focused plane. A z-spacing of less than 0.2 μm is required to limit errors to 3%. Errors increase to 20% for a more practical z-spacing of 0.5 μm.

However, one can limit the high error due to large z-spacing. As an alternative, one could base MT measurements on the sum of intensities in all *z*-planes:

(2) $$I_{sum}\left(h\right)=\frac{d}{\sigma \sqrt{2\pi }}\overset{5\;\mu m/d}{\underset{n=0}{\sum }}exp\left(-\frac{1}{2}{\left(\frac{h-nd}{\sigma }\right)}^{2}\right)$$

where *h* is the height of the MT in the scan volume and d is the z-spacing. A normalization factor is introduced in front of the summation sign. The oscillations in this sum-intensity are in general much smaller. For a z-spacing of 0.5 μm, errors are less than 1% for the center 4 μm of the scan volume. We therefore conclude that the intensity summation method gives the most precise measurement of MT intensity, and thus, number in the fission yeast cell.

For quantitative MT measurement, cells were imaged in three-dimension (3D) at 0.5 μm z-spacing and a 5-μm-thick section (to cover the 4 μm thickness of the cells), at 1 s exposure time for each section, and 30 s temporal resolution between each 3D stack. The cooled back-thinned CCD camera was set at high-precision mode and 1× binning to yield 0.0645 μm per pixel resolution.

**Figure 2:**
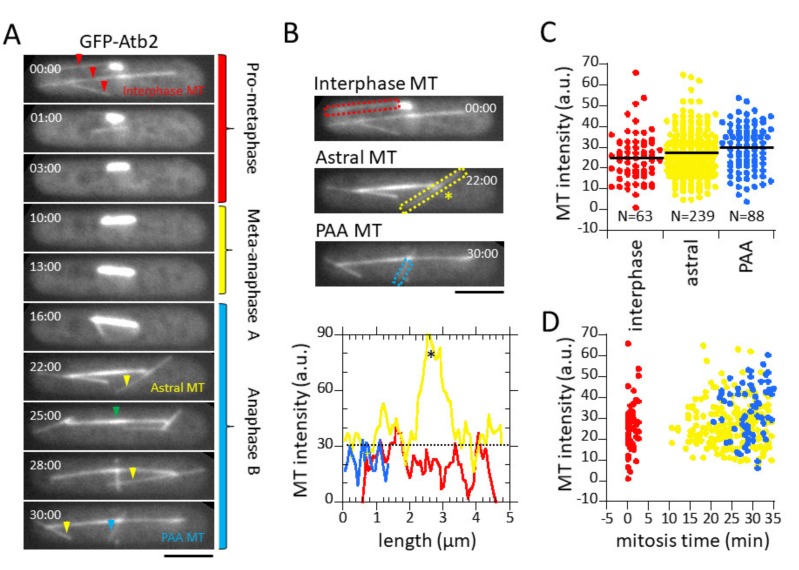
MT fluorescent intensity converts to MT number. (**A**) Shown is a time-lapse summation montage of a wild-type mitotic fission yeast cell expressing GFP-Atb2. Different microtubule structures emerged at different phases of the cell cycle. Interphase MTs (red arrow) begin to depolymerize at the start of mitosis. Prop-metaphase corresponds to the initial spindle formation where the spindle elongates to about ~3 µm long. Metaphase-anaphase A is where spindle length remains relatively unchanged. Anaphase B is where the spindle elongates to ~12 µm long then undergoes a breakdown. Early anaphase B marks the emergence of the astral MTs (yellow arrow) and late anaphase B marks the emergence of the PAA MTs (blue arrow). A clear central spindle region, or midzone, is evident during anaphase (green arrow). Note that while the spindle intensity changes throughout mitosis, the intensities of the interphase, astral, and PAA MTs remains relatively the same during the 30 min of imaging. Bar, 5 µm. (**B**) Plot showing line scans of the interphase, astral, and PAA MT bundles from the cell in Figure 2A. Note that the signal varies for each class of MTs, but all MTs have similar signal intensities. The asterisk * denotes the antiparallel MT overlap of the astral MT bundle. This overlap has 3× higher intensity compared to the distal MT regions. (**C**) Dot plot of fluorescence intensities of interphase, astral, and PAA microtubules. The three different MT structures have similar mean intensities of ~30 a.u. (**D**) Dot plot of fluorescence intensities of interphase, astral, and PAA microtubules versus mitosis time. The three different MT structures have similar mean intensity over the entire imaging period.

There was significant photobleaching during the ~30 min of imaging. We measured that photobleaching decreased the cytoplasmic GFP-Atb2 signal from 150 arbitrary units (a.u.) to 90 a.u., or ~40% from start to finish (Figure 1D). The average of the lowest detectable MT signals in cells is ~30 a.u., representing 1 MT (Figure 2B–D). Thus, a decrease from 150 a.u. to 90 a.u. over a period of 30 min imaging yields a maximum counting error of 2 MTs due to photobleaching. This is comparable to the variance of individual MT signals in a cell population.

### 2.4. Data Analysis

Images were viewed and analyzed with MetaMorph 6. The theoretical resolvable limit of our microscope setup is R = 0.61 λ/NA = 0.61 × 510 nm/1.45 ≈ 0.22 μm, or approximately the width of 4 pixels. In practice, the width of the fluorescence signal of the linear MT structure varied between 5–8 pixels. We defined a width of 6 pixels (for interphase MTs) and 10 pixels (for the mitotic spindle) as the scan width for measuring MT intensity along its long axis. Briefly, each 3D stack was projected by the summation of all planes onto a final image containing all MTs of the cell. All MT intensities are background corrected by subtracting the cellular background intensities prior to analysis. For the spindle, intensity line scans were performed on the entire lengths of the MT structures. This measurement was repeated for each time point throughout spindle elongation, effectively taking into account potential photobleaching over time. For interphase, astral, and PAA microtubules, intensity line scans were performed on ~1 μm regions at the distal tips of the MT structures.

Because the interphase, astral, and PAA microtubule structures consistently showed equal and lowest a.u. of intensity in the cell, we thus defined the mean intensity of the astral MTs as the minimum unit of fluorescence for MTs within each cell. Hoog et al., using electron tomography, previously showed that interphase fission yeast MT bundles are composed of primarily two individual MTs overlapping at the nuclear membrane [21]. Thus, we can normalize the intensity of interphase, astral, and PAA microtubules as representative of one individual MT.

We then normalized the spindle intensities to the minimum MT unit of fluorescence. Our normalized MT fluorescence represents relative changes in MT number in the cell throughout the cell cycle. Ding et al. had reported careful EM measurements of cross-sectional MT numbers on several wild-type fission yeast spindles at different phases of spindle elongation [20,23]. We therefore used the EM data as a calibration curve for our fluorescence measurement. We superimposed the EM values on our plots of normalized spindle intensities and found a near perfect fit with Ding et al. [20], but not Ward et al. [23].

For cellular αβ-tubulin concentration measurements, which have a unit of molarity, i.e., 1M = 6.02 × 10^23^ subunits/1 × 10^15^ μm^3^, we measured tubulin subunits and cell volume as follows. For cell volume, the length L and width 2R of each cell were measured. Fission yeast is rod-shaped, so each cell has a volume of:

(3) $$V=\pi R^{2}L-\left[\pi R^{2}2R-\frac{4}{3}\pi R^{2}\right]$$

For cellular tubulin subunits, we first measured the total fluorescence of the whole cell (I_cell_) and the total fluorescence of the MT structures (I_MT_) by circumscribing regions-of-interest around the whole cell and the MT structures, respectively (background intensities were subtracted). For the MT structures, the background was the adjacent cytoplasm. For the cell, the background was the adjacent empty extra-cellular space. We then calculated the ratio I_Cell_/I_MT_. We know that I_Cell_ = I_MT_ + I_Soluble_. We note that for this study, the soluble tubulin fraction represents cellular free tubulin not in polymer form. Therefore, if we can convert I_MT_ into an actual value for tubulin subunits, then I_Cell_ and I_Soluble_ can also be converted into tubulin subunits. The tubulin subunits inside each MT structure can be calculated by converting the total MT polymer lengths of the MT structures into subunits, i.e., a 13-protofilament MT has 1625 αβ-tubulin subunits per 1 µm length. Therefore, the total MT polymer lengths inside the MT structures, such as interphase MT bundles or the mitotic spindle, can be used to calculate the total tubulin subunits in each MT structure. The cellular tubulin concentration is then calculated based on the total tubulin subunits and cell volume. Total MT polymer lengths for the spindle were calculated by multiplying the number of MTs emanating from each SPB with the half-length of the spindle for each time point.

## 3. Results

### 3.1. Sum of z-Sections and 0.5 µm z-Spacing Yield Practical Quantitative Measurements of Microtubules

Limitations in quantifying fluorescent intensities in living cells include photobleaching and sample movement away from the focus plane. Photobleacing, due to time-dependent exposure to excitation lights, contributes error to the intensity measurement particularly at later time points. Photobleaching can be largely minimized with efficient and sensitive imaging methods, such as the spinning disk confocal microscope coupled to a high quantum yield, high signal-to-noise cooled CCD camera [20]. Sample movement away from the focus plane, due to the dynamic nature of subcellular structures, also contributes error to the intensity measurement, i.e., intensity fluctuates if an object such as a MT moves away from the focus plane. In theory, performing fast and fine optical z-sectioning through the cell will capture the in focus plane. In practice, there is a sweet spot speed and z-section sampling which should give both low intensity error and high spatial-temporal resolution.

We measured the point spread function (PSF) of our optical set-up and derived the fluorescent intensity errors due to sample movement (see Methods). From the measured PSF, we derived the intensity error due to sample movement out of the focus plane for a 5-µm-thick object sampled at 0.1, 0.2, 0.5, and 1 µm z-spacing. Maximum intensities were observed when an object was in focus. Minimum intensities were observed when an object was exactly half-way between two focused planes. The intensity error increased from 1% at 0.1 µm, to 3.5% at 0.2 µm, to 20% at 0.5 µm, and up to 60% at 1 µm z-spacing (Figure 1A). We next derived the intensity error of sums of all planes for the different z-spacing. The intensity error due to sample movements decreased to 0% when all frames at each 0.1, 0.2, or 0.5 µm z-spacing were summed (Figure 1A). Therefore, the sum of all z-planes yields more precise fluorescence quantification.

We next determined the z-spacing as a function of temporal resolution. The percent intensity error follows the function:

(4) $$E\left(z\right)=\frac{I{\left(z\right)}_{Focus}-I{\left(z\right)}_{Unfocus}}{I{\left(z\right)}_{Focus}}$$

where *E* is the intensity error (%), *z* is the z-spacing (µm), and *I_Focus_* and *I_Unfocus_* are the intensity at the focus and out-of-focus plane. The total number of z-planes in a stack and with that the total acquisition time required to capture a given thickness, decreases with increasing z-spacing as follows:

(5) $$T\left(z\right)=\frac{D}{z}+1$$

where *T* is total time, *D* is the thickness of the sampled object (µm), and *z* is the z-spacing (µm). We defined the intersection between the two curves of the functions *E(z)* and *T(z)* as the optimal imaging condition where intensity error and acquisition time are minimized.

We found that in the context of our microscope set-up, the practical z-spacing is 0.5 µm (Figure 1A,C), corresponding to 11 optical sections covering 5 µm thickness (fission yeast is 4 µm thick). We conclude that: (1) the smaller the z-spacing, the less intensity error would be generated; (2) the sum of all z-planes at each z-spacing yields an intensity measurement with less error than maximum-projection images; and (3) a z-spacing of 0.5 µm is the practical spacing for quantitative fluorescence measurements. In other words, 0.5 µm z-spacing is the practical choice for which the intensity error is small and the stack acquisition time is short.

**Figure 3:**
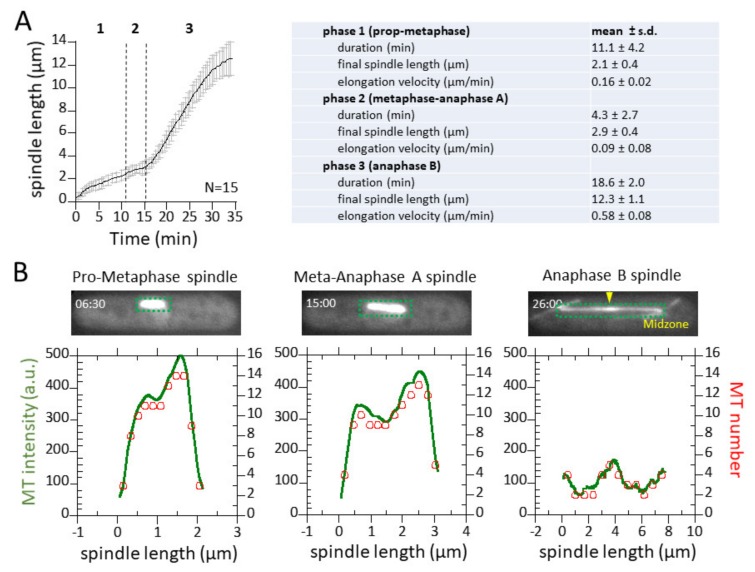
Spindle dynamics and corresponding MT intensity and number. (**A**) Plot of spindle length versus time. Shown are mean length ± s.d. for 15 wild-type fission yeast cells. Summary table of spindle dynamics for 15 wild-type fission yeast cells. (**B**) Representative plots of spindle fluorescence intensities for three different time points or spindle lengths (green). The intensities are converted to MT number (red) by dividing the total intensity over the mean intensity of one MT.

**Figure 4:**
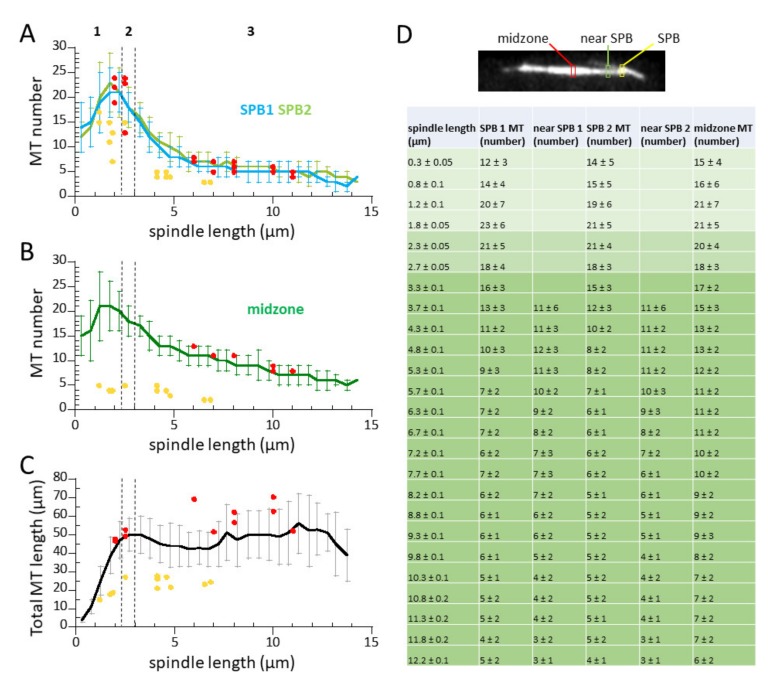
MT number changes throughout mitosis. (**A**) Plot of MT number versus spindle length. Shown are mean MT number ± s.d. emanating from the two SPBs (green/blue). Red dots and yellow dots represent MT number versus spindle length at the SPB measured by EM by Ding et al. [20] and Ward et al. [23], respectively. (**B**) Plot of MT number versus spindle length. Shown are mean MT number ± s.d. at the spindle midzone region (green). Red dots and yellow dots represent MT number versus spindle length at the spindle midzone measured by EM by Ding et al. [20] and Ward et al. [23], respectively. Note that the MT number obtained from quantitative fluorescent live-cell imaging (this study) matches closely with those obtained by EM by Ding et al. [20]. (**C**) Plot of total MT polymer length versus spindle length. Red dots and yellow dots represent total MT length versus spindle length measured by EM by Ding et al. [20] and Ward et al. [23], respectively. Note that even as MT number decreases as mitosis progresses, cells maintain a constant total MT polymer length. (**D**) Summary table of spindle MT length and number throughout mitosis. Image of mitotic cell with labels of regions of MT measurement. SPBs are defined arbitrarily as SPB 1 has the higher signal of the two SPBs.

We also quantified the photobleaching resulting from long-term imaging (see Methods). The GFP-Atb2 signal decreased from 150 a.u. to 90 a.u., or ~40%, over a period of 30 min (Figure 1D). The mean intensity of cytoplasmic MTs is ~30 a.u. (see Figure 2). This indicates a potential counting error of ~2 MTs at the end of 30-min imaging. This error is within the range of spindle signal variation (see Figure 3 and Figure 4). In addition, photobleaching of GFP-Atb2 is not expected to affect the MT length measurement.

### 3.2. Microtubule Fluorescence is a Direct Measurement of Microtubule Number

We imaged wild-type living fission yeast cells expressing GFP-Atb2 at the practical settings for quantitative measurements of microtubules throughout the cell cycle (see Section 3.1 above). Cells were imaged from the interphase to mitosis transition (G_2_ to M) to capture all classes of microtubule structures, i.e., interphase MTs, spindle MTs, astral MTs, and PAA microtubules. A montage of a representative wild-type cell undergoing spindle elongation at mitosis, and the diverse MT structures—interphase, astral, PAA microtubules, and the spindle structure—is shown (Figure 2A).

The interphase, astral, and PAA microtubules were very dynamic, and showed similar fluorescent intensities. For each cell, we quantified the intensities of the interphase, astral, and PAA microtubule structures (see Methods). Line scans revealed that interphase, astral, and PAA MTs have similar fluorescent signal intensities throughout their long axis (Figure 2B). While these three different classes of MTs occurred at different times during the cell cycle, they showed similar mean ± standard deviation (s.d.) intensities of 31 ± 12 a.u. (interphase MTs), 35 ± 13 a.u. (astral MTs), and 36 ± 11 a.u. (PAA MTs) (Figure 2C,D). Because fluorescent intensity is a function of polymer number, our results indicated that interphase, astral, and PAA microtubule bundles all have a similar number of MTs. Further, the intensities of these different classes of MTs were always the lowest intensities observed in the cell, as compared to the spindle intensities, indicating that they represented the smallest, or one fluorescence unit of MT number. We previously reported that interphase and astral MT bundles are organized with their minus ends crosslinked at the cell middle or the spindle pole body (SPB), respectively, by the protein Ase1, and their dynamic distal plus ends interacting with the cell cortex [28]. In addition, Hoog et al. showed by electron microscopy (EM) that each fission yeast interphase MT bundle is composed of a few distinct MTs [21]. In particular, the distal plus ends facing opposite cell tips are generally composed of a single MT [21]. Thus, our MT intensity of ~34 a.u. (the average of interphase, astral, and PAA MT signal) likely represents 1 distinct MT and serves as a calibration for all other MT signals. Each interphase MT, astral MT, and PAA MT structure are thus primarily composed of single MTs crosslinked at their minus ends.

### 3.3. Spindle Elongation Exhibits 3-Phase Elongation Kinetics and Dynamic Microtubules

We imaged 15 mitotic events. Fission yeast mitosis is remarkably consistent [14,28], showing the typical three-phase spindle elongation kinetics, representing pro-metaphase, metaphase-anaphase A, and anaphase B (Figure 3A). We measured the complete spindle intensity through time. For each time point, the measured MT fluorescent intensity along the spindle length was normalized to the fluorescent unit representing one MT (34 a.u. is the average intensity of the individual interphase, astral, and PAA MTs). The normalization procedure converted absolute MT fluorescent intensities into relative MT numbers along the length of the spindle. For example, for the cell shown in Figure 2A, at the 6.5 min time point (pro-metaphase), the spindle reached 2 µm in length, and had a maximum intensity of 500 a.u., which when normalized by dividing by 34 a.u. yielded 16 MTs (Figure 3B). Effectively, all intensities at all spatial-temporal points can be normalized in this way to give relative measurements of MT numbers. 

Figure 3A,B illustrates the dynamic changes of spindle MT numbers at different spindle elongation phases. Pro-metaphase was characterized by a growing spindle with bi-modal MT distribution, where the middle region of the bipolar spindle showed a decrease in MT number compared to the regions at the SPBs (Figure 3B, time = 6.5 min). We interpret this exclusion zone as the zone of MT and chromosome interaction. Meta-anaphase A showed a similar profile of spindle MTs compared to pro-metaphase (Figure 3B, time = 15 min). However, we began to observe a decreased number of MTs at the spindle pole region. Anaphase B clearly showed the spindle midzone region, which had the highest spindle MT number due to overlapping MTs emanating from opposite spindle pole bodies (SPBs) (Figure 3B, time = 26 min). Our conversion of fluorescent intensities into MT number, and the subsequent interpretation of MT organization, are only possible with EM foundational work describing the interphase MT and spindle structures of fission yeast [20,21,23].

### 3.4. Microtubule Number Decreases, but Total Microtubule Polymer Length Remains Constant During Mitosis

We then measured the complete spindle MT number throughout mitosis. During the pro-metaphase (phase 1), ~12 MTs emanated from each SPB at the onset of spindle formation to form the spindle structure. The number of MTs at each SPB increased to the maximum 23 ± 6 at the end of the pro-metaphase (Figure 4A,D). Microtubule numbers emanating from both SPBs were nearly identical throughout mitosis. During metaphase-anaphase A (phase 2), the spindle MT number began to decrease from the maximum found at the end of prophase to 17 ± 3 at the end of metaphase-anaphase A (Figure 4A,D).

During anaphase B (phase 3), the number of MTs emanating from the two SPBs continued to decrease. At the moment just before spindle breakdown at the end of anaphase B, there were approximately 4 ± 1 MTs emanating from each SPB (Figure 4A,D). The MT number found at the spindle midzone follows a similar trend as MTs emanating from the SPBs (Figure 4B). However, at the start of anaphase, the midzone MT number began to increase compared to the MT number at the SPBs, a clear indication of the overlapping MTs indicative of the midzone (Figure 4B). Figure 4D summarizes the number of MTs found in the dynamic spindle of wild-type fission yeast.

Noteworthy, two previous EM studies reconstructed the number and lengths of spindle MTs for wild-type fission yeast with spindle lengths ranging from 1–11 µm [20,23]. We overlaid these static data points onto our dynamic measurements and found an exact match to the Ding et al. data (Figure 4A,B, red dots). However, the Ward et al. data were generally lower than those of Ding et al. and ours (Figure 4A,B, yellow dots). We conclude that our fluorescent imaging and analysis method has the ability to quantify individual MTs when cross-referenced with EM data, even when the MT number in a bundle is not spatially resolved by optical microscopy. Further, our quantitative fluorescent imaging method extends on current EM methods and enables a direct read out of MT number and distribution in individual living cells with high temporal resolution.

### 3.5. Tubulin Concentrations in Interphase and Mitotic Cells

What is the αβ-tubulin concentration in a living fission yeast cell? Total tubulin subunits in a cell are composed of the cytoplasmic fraction plus the fraction that has incorporated into the MT polymer. As we now have a method to quantify the polymerized fraction, we should be able to use the fluorescent intensity of the whole cell to derive the soluble fraction, i.e., I_Cell_ = I_MT_ + I_Soluble_. As fission yeast has a closed mitosis, where the nuclear membrane does not breakdown, the soluble tubulin indicates tubulin not in MT form, in the whole cell irrespective of cytoplasmic or nucleoplasmic localization. We measured the fluorescent intensities I_Cell_ and I_MT_ of mitotic wild-type cells as boxed regions around the whole cell and spindle (see Methods). We found that the total fluorescent intensity of the cell, I_Cell_, remained constant throughout mitosis during metaphase and anaphase, and the average ratio of cell intensity to spindle intensity, I_Cell/_I_MT_, was 3.88 ± 0.58, i.e., the total cell has ~4× more total tubulin subunits than that of the spindle alone. Because each spindle has 48 ± 5 µm of total MTs (Figure 4C), each cell, therefore, has the equivalent of 186 ± 15 µm (3.88 × 48 µm) of MTs. Given that a MT has 13 protofilaments, and each 1 µm of MT has 1625 αβ-tubulin subunits, then each fission yeast cell has 3.0 ± 0.2 × 10^5^ αβ-tubulin subunits (186 µm × 1625 subunits/µm) at mitosis. We calculated the average volume of these cells to be 94 ± 13 µm^3^ (see Methods). Therefore, the molar concentration of αβ-tubulin, the fission yeast cell during mitosis, is 5.35 ± 0.55 µM, of which three-quarters is soluble tubulin and one-quarter is in the MT polymer in the spindle (Table 1).

Fission yeast has a characteristic unipolar growth during G_1_-S, where cells are relatively short after exiting cytokinesis and septation, and grow at their old end. At G_2_, fission yeast switches to bipolar growth, a process termed new-end-take-off (NETO). The length of fission yeast serves as a good indicator of cell cycle stages [29]. Throughout interphase, fission yeast has an average of 4 ± 1 discrete bundles of MTs, with each bundle having an overlapping medial region [6,8,28,30]. We measured the average tip-to-tip bundle length to be 5.3 ± 1.6 µm and 8.4 ± 2.9 µm, and the average overlapping region to be 1.0 ± 0.4 µm and 1.2 ± 0.8 µm, for pre- and post-NETO interphase cells, respectively. In a similar fashion to the mitotic cells, we measured the molar concentration of αβ-tubulin, the fission yeast cell during interphase. We found that the total fluorescent intensity of the cells, I_Cell_, increased throughout interphase. However, the ratio of cell intensity to MT bundle intensities remained constant throughout G_1_-S and G_2_, with the average ratio of cell intensity to MT bundle intensities, I_Cell_/I_MT_, as 2.85 ± 0.80 for G_1_-S and 2.96 ± 0.27 for G_2_ interphase cells, i.e., the total cell has ~3× more total tubulin subunits than that of the combined interphase MT polymers (Table 1). By the same reasoning used to calculate tubulin concentrations for mitotic cells, pre-NETO interphase cells have an average MT polymer length of 23 ± 4 µm and post-NETO cells have an average MT polymer length of 41 ± 5 µm. Thus, G_1_-S interphase fission yeast cells have 1.06 ± 0.30 × 10^5^ αβ-tubulin subunits, and a total tubulin concentration of 4.04 ± 0.24 µM; and G_2_ interphase cells have 1.97 ± 0.24 × 10^5^ αβ-tubulin subunits and total tubulin concentration of 3.83 ± 0.32 µM. These values are similar to previously reported values using quantitative mass spectrometry [19,22]. We conclude that interphase cells maintained a relatively constant molar concentration of tubulin, of which two-thirds is in αβ-tubulin form in the cytoplasm and one-third is in MT polymer form (Table 1).

We note that our imaging method systematically underestimates the interphase cellular αβ-tubulin concentrations, but not the mitosis cellular tubulin concentrations. Careful EM reconstruction of the fission yeast mitotic spindles showed that all spindle MTs emanated from the SPBs [20,23]. As our fluorescence measurement of spindle MTs matched those of EM data from Ding et al. (Figure 4A,B), our fluorescence measurement for spindle MTs is therefore exact. In contrast, there are satellite-MTOCs that are recruited to the lattice of a pre-existing interphase MT, where they nucleate new short MTs [31,32,33]. In addition, EM tomography of interphase fission yeast MTs also showed short MTs attached to the primary MT [21]. These findings suggest that our optical method would fail to detect sub-resolution short MTs, i.e., MTs shorter than ~0.3–0.5 µm. Sub-resolution short MTs comprise ~25% of the total MT polymer length at interphase [21]. This suggests that we may have underestimated the interphase cellular tubulin concentrations by ~33% (25% short MTs /75% long MTs = 33%), i.e., instead of the measured ~4 µM, the correct interphase concentration may be ~5.32 µM (4 µM × 1.33), or equal to our measured mitosis concentration of 5.35 ± 0.55 µM (Table 1). This implies that the total cellular tubulin concentration remains constant throughout the cell cycle, and the transition from interphase to mitosis sees an up regulation in total MT polymer.

## 4. Discussion

In the current study, we presented a method for quantitative fluorescent imaging and analysis of MTs in living fission yeast cells. This method, coupled with reported EM data of MT organization and number [20,21,23], provides a dynamic description of MTs throughout the cell cycle. We found that: (1) a z-spacing of 0.5 µm and the sum of all z-sections provides a practical quantification of fluorescent signals, with a resolution of 1–2 MTs (Figure 1, Figure 2 and Figure 3); (2) during mitosis, each SPB can nucleate ~23 individual MTs (Figure 4 and Table 1)); and (3) the cellular αβ-tubulin concentration is ~5 µM throughout the cell cycle (Table 1).

### 4.1. Cellular αβ-Tubulin Concentrations

We reported here in vivo cellular αβ-tubulin concentrations of ~5 µM during interphase and mitosis for fission yeast observed at room temperature (Table 1). Interestingly, one-third of the interphase tubulin pool exists in polymer form and one-quarter of the mitotic tubulin pool exists in polymer form. Previous works using fluorescently-tagged tubulin injected into mammalian tissue culture cells, while unable to quantify the exact tubulin concentrations, reported that the fraction of tubulin in polymer form to be three-quarters for interphase and two-thirds for mitotic cells at 37 °C [34,35]. However, when these mammalian cells were observed at room temperature, 23 °C, the tubulin fraction in polymer form reduced to 30% at mitosis, close to the 26% observed in fission yeast at the same temperature. This similarity between mammalian cells and fission yeast highlights conserved core mechanisms that regulate steady-state tubulin polymerization dynamics and that temperature has a profound influence on MTs [36].

The phase diagram of MT growth for purified porcine tubulin in vitro at varying concentrations and temperatures was previously reported [36]. While care must be taken when trying to compare in vivo to in vitro experiments, we note that an in vitro tubulin concentration of ~5 µM is too low to support spontaneous MT nucleation and polymerization in vitro at 23 °C [36]. This suggests that perhaps ~5 µM of tubulin in vivo may also be too low to support spontaneous MT nucleation and polymerization and, therefore, nucleating seeds such as γ-tubulin complexes become essential for nucleating and polymerizing MTs within the cell.

### 4.2. Differences in Mass Spectrometry, Electron Microscopy, and Fluorescent Imaging in Quantifying Microtubules

Our measurement of MT number throughout the cell cycle compares well with previous measurements using EM [20,21,23]. In particular, our spindle MT measurement agrees completely to those seen in the EM work of Ding et al. [20], but not of the EM work of Ward et al. [23]. Ward et al. reported that their EM technique introduced curvatures in the spindle which were not seen with live-cell imaging [23]. In addition, the number of MTs present in the spindle reported by Ward et al. [23] is consistently less than that of Ding et al. [20]. In this context, the EM sample preparation methods of Ding et al. likely preserve the true state of MTs in fission yeast [20].

Our measurement of cellular tubulin number and tubulin concentration in fission yeast also compares well with previous measurements using quantitative mass spectrometry [19,22]. Differences observed may be due to measuring single cells in the case of fluorescent imaging and averaging population of cells in the case of mass spectrometry.

## 5. Conclusions

With the increasing use of fluorescent live-cell imaging and the emerging system biology approach, modern cell biology is becoming quantitative. Methods to quantify concentrations, localization, and dynamics of fluorescently tagged proteins in individual living cells will be essential for a molecular understanding of their function. Our simple method to quantify the exact changes in MT dynamics in the fission yeast is anticipated to be of general applicability to other proteins. However, we note also that the precision of quantitative methods in optical microscopy requires complementary and correlative methods in electron microscopy. For example, our measured spindle MT’s relative fluorescence was made precise by correlating with EM reconstructions of spindle MTs [20]; and our measured interphase MT number is similar to that observed by EM tomography [21]. Clearly, slow-but-precise EM complements well fast-and-noisy light microscopy. In combination, the two methods can give precise parameters on steady-state microtubule dynamics.

## Figures and Tables

**Table 1:** Tubulin concentrations in interphase and mitotic wild-type cells.

	Interphase (G_1_-S)	Interphase (G_2_)	Mitosis (M)
Cell length (μm)	7.38 ± 0.43	12.82 ± 1.16	14.62 ± 1.46
Cell width (μm)	2.94 ± 0.14	3.03 ± 0.11	2.96 ± 0.13
Cell volume (μm^3^)	43.36 ± 3.35	85.32 ± 11.21	93.76 ± 13.10
Number of MT bundles	3.71 ± 0.49	4.11 ± 0.60	vary, *
Tip-to-tip length of bundles	5.29 ± 1.55	8.48 ± 2.92	vary, *
Total MT polymer length (μm)	22.82 ± 3.70	40.94 ± 5.00	47.86 ± 4.59
Fraction of tubulin in MT polymer (%)	35	34	26
Fraction of tubulin in cytoplasm (%)	65	66	74
Total tubulin subunit per cell	1.06 ± 0.30 × 10^5^	1.97 ± 0.24 × 10^5^	3.02 ± 0.45 × 10^5^
Total tubulin concentration in cell (μM)	4.04 ± 0.24	3.83 ± 0.32	5.35 ± 0.55
Tubulin concentration in MT (μM)	1.41 ± 0.24	1.30 ± 0.32	1.39 ± 0.55
Tubulin concentration in cytoplasm (μM)	2.63 ± 0.24	2.53 ± 0.32	3.96 ± 0.55
§ Corrected total tubulin subunit per cell	1.41 ± 0.40 × 10^5^	2.62 ± 0.32 × 10^5^	
§ Corrected total tubulin concentration in cell (μM)	5.37 ± 0.32	5.09 ± 0.43	
§ Corrected concentration in MT (μM)	1.88 ± 0.32	1.73 ± 0.43	
§ Corrected concentration in cytoplasm (μM)	3.49 ± 0.32	3.36 ± 0.43	
Cells measured (N)	7	9	6

G1-S interphase cells are short 7–9 μm pre-NETO cells; G2 interphase cells are long 9–14 μm post-NETO cells [29]. § Note: Our optical microscopy method cannot resolve short MTs <0.5 μm in length. Therefore, we may have systematically underestimated the tubulin concentrations in interphase cells by ~33% (see Discussion). Thus, we also report the corrected values. * Note: Values for mitotic spindle MTs are reported in Figure 4D. Values are mean ± s.d.

## References

[1] Hayles J., Nurse P. (2001). A journey into space. Nat. Rev. Mol. Cell Biol..

[2] Nurse P. (1975). Genetic control of cell size at cell division in yeast. Nature.

[3] Wu J.Q., Kuhn J.R., Kovar D.R., Pollard T.D. (2003). Spatial and temporal pathway for assembly and constriction of the contractile ring in fission yeast cytokinesis. Dev. Cell.

[4] Wu J.Q., Pollard T.D. (2005). Counting cytokinesis proteins globally and locally in fission yeast. Science.

[5] Ding D.Q., Chikashige Y., Haraguchi T., and Hiraoka Y. (1998). Oscillatory nuclear movement in fission yeast meiotic prophase is driven by astral microtubules, as revealed by continuous observation of chromosomes and microtubules in living cells. J. Cell Sci..

[6] Drummond D.R., Cross R.A. (2000). Dynamics of interphase microtubules in *Schizosaccharomyces pombe*. Curr. Biol..

[7] Sagolla M.J., Uzawa S., Cande W.Z. (2003). Individual microtubule dynamics contribute to the function of mitotic and cytoplasmic arrays in fission yeast. J. Cell Sci..

[8] Tran P.T., Marsh L., Doye V., Inoue S., Chang F. (2001). A mechanism for nuclear positioning in fission yeast based on microtubule pushing. J. Cell Biol..

[9] Daga R.R., Chang F. (2005). Dynamic positioning of the fission yeast cell division plane. Proc. Natl. Acad. Sci. USA.

[10] Tolic-Norrelykke I.M., Sacconi L., Stringari C., Raabe I., Pavone F.S. (2005). Nuclear and division-plane positioning revealed by optical micromanipulation. Curr. Biol..

[11] Brunner D., Nurse P. (2000). CLIP170-like tip1p spatially organizes microtubular dynamics in fission yeast. Cell.

[12] Mata J., Nurse P. (1997). tea1 and the microtubular cytoskeleton are important for generating global spatial order within the fission yeast cell. Cell.

[13] Piel M., Tran P.T. (2009). Cell shape and cell division in fission yeast. Curr. Biol..

[14] Nabeshima K., Nakagawa T., Straight A.F., Murray A., Chikashige Y., Yamashita Y.M., Hiraoka Y., Yanagida M. (1998). Dynamics of centromeres during metaphase-anaphase transition in fission yeast: Dis1 is implicated in force balance in metaphase bipolar spindle. Mol. Biol. Cell.

[15] Tolic-Norrelykke I.M., Sacconi L., Thon G., Pavone F.S. (2004). Positioning and elongation of the fission yeast spindle by microtubule-based pushing. Curr. Biol..

[16] Hagan I.M. (1998). The fission yeast microtubule cytoskeleton. J. Cell Sci..

[17] Samejima I., Miller V.J., Rincon S.A., Sawin K.E. (2010). Fission yeast Mto1 regulates diversity of cytoplasmic microtubule organizing centers. Curr. Biol..

[18] Sawin K.E., Tran P.T. (2006). Cytoplasmic microtubule organization in fission yeast. Yeast.

[19] Carpy A., Krug K., Graf S., Koch A., Popic S., Hauf S., Macek B. (2014). Absolute proteome and phosphoproteome dynamics during the cell cycle of *Schizosaccharomyces pombe* (Fission Yeast). Mol. Cell Proteomics.

[20] Ding R., McDonald K.L., McIntosh J.R. (1993). Three-dimensional reconstruction and analysis of mitotic spindles from the yeast, *Schizosaccharomyces pombe*. J. Cell Biol..

[21] Hoog J.L., Schwartz C., Noon A.T., O’Toole E.T., Mastronarde D.N., McIntosh J.R., Antony C. (2007). Organization of interphase microtubules in fission yeast analyzed by electron tomography. Dev. Cell.

[22] Marguerat S., Schmidt A., Codlin S., Chen W., Aebersold R., Bahler J. (2012). Quantitative analysis of fission yeast transcriptomes and proteomes in proliferating and quiescent cells. Cell.

[23] Ward J.J., Roque H., Antony C., Nedelec F. (2014). Mechanical design principles of a mitotic spindle. eLife.

[24] Moreno S., Klar A., Nurse P. (1991). Molecular genetic analysis of fission yeast *Schizosaccharomyces pombe*. Methods Enzymol..

[25] Tran P.T., Paoletti A., Chang F. (2004). Imaging green fluorescent protein fusions in living fission yeast cells. Methods.

[26] Maundrell K. (1990). *nmt1* of fission yeast. A highly transcribed gene completely repressed by thiamine. J. Biol. Chem..

[27] Inoue S., Inoue T. (2002). Direct-view high-speed confocal scanner: The CSU-10. Methods Cell Biol..

[28] Loiodice I., Staub J., Setty T.G., Nguyen N.P., Paoletti A., Tran P.T. (2005). Ase1p organizes antiparallel microtubule arrays during interphase and mitosis in fission yeast. Mol. Biol. Cell.

[29] Mitchison J.M., Nurse P. (1985). Growth in cell length in the fission yeast *Schizosaccharomyces pombe*. J. Cell Sci..

[30] Yamashita A., Sato M., Fujita A., Yamamoto M., Toda T. (2005). The roles of fission yeast ase1 in mitotic cell division, meiotic nuclear oscillation, and cytokinesis checkpoint signaling. Mol. Biol. Cell.

[31] Janson M.E., Loughlin R., Loiodice I., Fu C., Brunner D., Nedelec F.J., Tran P.T. (2007). Crosslinkers and motors organize dynamic microtubules to form stable bipolar arrays in fission yeast. Cell.

[32] Janson M.E., Setty T.G., Paoletti A., Tran P.T. (2005). Efficient formation of bipolar microtubule bundles requires microtubule-bound γ-tubulin complexes. J. Cell Biol..

[33] Samejima I., Lourenco P.C., Snaith H.A., Sawin K.E. (2005). Fission yeast mto2p regulates microtubule nucleation by the centrosomin-related protein mto1p. Mol. Biol. Cell.

[34] Zhai Y., Borisy G.G. (1994). Quantitative determination of the proportion of microtubule polymer present during the mitosis-interphase transition. J. Cell Sci..

[35] Zhai Y., Kronebusch P.J., Simon P.M., Borisy G.G. (1996). Microtubule dynamics at the G2/M transition: Abrupt breakdown of cytoplasmic microtubules at nuclear envelope breakdown and implications for spindle morphogenesis. J. Cell Biol..

[36] Fygenson D.K., Braun E., Libchaber A. (1994). Phase diagram of microtubules. Phys. Rev. E Stat. Phys. Plasmas Fluids Relat. Interdiscip. Top..

